# Pituitary Androgen Receptor Signalling Regulates Prolactin but Not Gonadotrophins in the Male Mouse

**DOI:** 10.1371/journal.pone.0121657

**Published:** 2015-03-23

**Authors:** Laura O’Hara, Michael Curley, Maria Tedim Ferreira, Lyndsey Cruickshanks, Laura Milne, Lee B. Smith

**Affiliations:** Medical Research Council Centre for Reproductive Health, University of Edinburgh, Edinburgh, United Kingdom; Baylor College of Medicine, UNITED STATES

## Abstract

Production of the androgen testosterone is controlled by a negative feedback loop within the hypothalamic-pituitary-gonadal (HPG) axis. Stimulation of testicular Leydig cells by pituitary luteinising hormone (LH) is under the control of hypothalamic gonadotrophin releasing hormone (GnRH), while suppression of LH secretion by the pituitary is controlled by circulating testosterone. Exactly how androgens exert their feedback control of gonadotrophin secretion (and whether this is at the level of the pituitary), as well as the role of AR in other pituitary cell types remains unclear. To investigate these questions, we exploited a transgenic mouse line (Foxg1^Cre/+^; AR^fl/y^) which lacks androgen receptor in the pituitary gland. Both circulating testosterone and gonadotrophins are unchanged in adulthood, demonstrating that AR signalling is dispensable in the male mouse pituitary for testosterone-dependent regulation of LH secretion. In contrast, Foxg1^Cre/+^; AR^fl/y^ males have a significant increase in circulating prolactin, suggesting that, rather than controlling gonadotrophins, AR-signalling in the pituitary acts to suppress aberrant prolactin production in males.

## Introduction

Production of testosterone by testicular Leydig cells is tightly regulated by the hypothalamic-pituitary-gonadal (HPG) axis, forming a homeostatic negative feedback loop. Gonadotrophin-releasing hormone (GnRH), secreted by the hypothalamus, stimulates secretion of luteinising hormone (LH) from the pituitary, which stimulates testosterone production by Leydig cells. Testosterone then feeds back to the hypothalamic-pituitary element to negatively regulate further LH secretion in a dose-dependent manner [1, 2]. Development of this negative feedback loop is essential for homeostatic maintenance of circulating testosterone concentrations in males; exactly how this is affected is not entirely understood, nevertheless androgen feedback at the level of both the hypothalamus and the pituitary has become the widely accepted paradigm.

Androgens impart their effects on transcription by binding to the androgen receptor (AR) [3]. AR is found in both the hypothalamus and pituitary in the mouse [4] suggesting androgens are able to feedback at both locales to regulate circulating gonadotrophin levels. Indeed in Testicular Feminisation (*Tfm*) mice (mice with a null allele of AR and little testosterone production [5]), levels of serum LH and follicle stimulating hormone (FSH) are increased [6]. LH levels are also increased in humans with complete androgen insensitivity syndrome (CAIS) in agreement with a general role for AR negative feedback in regulation of gonadotrophins [7]. Unfortunately, mouse models of global AR ablation add little to clarify how androgen feedback acts on the HPG axis as the loss of AR throughout all cells of the HPG axis prevents assignment of specific functional roles for androgen signalling in each cell type. Other attempts to identify the specific site of action of feedback by testosterone on gonadotrophin secretion have been widely explored and reveal a more complex system than androgens simply binding to AR throughout the HPG axis. In rams and rhesus monkeys, testosterone acts by decreasing the pulse rate of GnRH at the level of the hypothalamus but has negligible pituitary effects [1]. In addition, data from clinical studies on patients with hypogonadotrophic hypogonadism suggest that, while testosterone feedback is required for control of GnRH release by the hypothalamus, aromatisation of testosterone to estradiol is sufficient for control of LH secretion by the pituitary [8–10], suggesting the primary site of androgen feedback in human males is the hypothalamus.

However, there is some evidence for a role for androgen signalling in the pituitary. Testosterone has been shown to suppress LH secretion in rat gonadotroph cultures [11, 12] and AR can suppress transcription of the LHβ subunit by interacting with SF-1 [13] suggesting a key role for androgen signalling exists at the level of the pituitary, at least in rodents.

Taken together, these data paint a confusing picture of the role of AR signalling in the pituitary that is inconsistent with the widely accepted paradigm. Intriguingly, AR is widely expressed in several other cell populations within the pituitary in addition to the gonadotrophs [14, 15], suggesting that AR signalling may have further significant roles in the male pituitary, that to date remain undescribed. Indeed data from a recent study has suggested a new role for pituitary AR in control of glucocorticoid action [4]. This finding alone clearly demonstrates that our current understanding of the function of androgen signalling in the pituitary remains incomplete.

In a previous study, we utilised Foxg1-Cre to ablate AR from the caput epididymal epithelium, demonstrating that local ablation of AR perturbed initial segment development, an observation confirmed by a second group using an epididymis-specific Cre Recombinase line [16]. Further investigation revealed that Foxg1-Cre is also active in the developing Rathke’s pouch (the anlage of the pituitary) but not the hypothalamus [17–19] in this model, suggesting we had serendipitously generated a mouse model also lacking pituitary AR. Intriguingly, circulating testosterone concentrations in this mouse model are unaffected by AR ablation [16], an observation inconsistent with the currently accepted negative-feedback paradigm. In this study, we describe the detailed characterisation of this unique model, revealing previously unknown functions for androgen signalling in the male pituitary with implications for our understanding of the control of the male HPG axis.

## Methods

### Targeted ablation of AR from the pituitary using *Foxg1*-Cre mice

Mice in which AR has been ablated selectively from the pituitary were generated using Cre/*loxP* technology. Male congenic 129svev mice carrying a carrying a random insertion of *Foxg1*-Cre [19] were mated to C57BL/6J female mice homozygous for a floxed AR [20]. Male offspring were either the Foxg1^Cre/+^; AR^fl/y^ mice with androgen receptor ablation or Foxg1^+/+^; AR^fl/y^ ‘control’ littermates. Foxg1^+/+^; AR^+/y^ mice were also generated by mating *Foxg1*-Cre stud males to C57BL/6J females to confirm that expression of Cre alone did not induce a phenotype. Sex and genotype ratios were all identified at the expected Mendelian ratios. Mice were fed a soya-free diet. All mice were bred under standard conditions of care and use under licenced approval from the UK Home Office (60/4200).

### PCR genotyping of mice

Mice were genotyped for inheritance of Cre Recombinase as previously described [16]. PCR amplification products were resolved using a QiaXcel capillary system (Qiagen, UK). An amplicon of 102 bp indicated inheritance of the Cre Recombinase transgene.

### Recovery of pituitaries

Mice were euthanised by inhalation of carbon dioxide and subsequent cervical dislocation (adults) or injection of sodium pentobarbital (neonates) at 10:00h. Pituitaries were either fixed in Bouin’s fixative (Clin-Tech, Guildford, UK) or 10% neutral buffered formalin (NBF) for 6 hours or frozen on dry ice before being transferred to a -80°C freezer for storage. Fixed tissues were processed and embedded in paraffin wax, and 5μm sections were used for histological analysis. Sections of testis were stained with haematoxylin and eosin using standard protocols.

### Immunohistochemistry

Immunostaining was performed either by a single antibody colourimetric (DAB) immunostaining method, as described previously [16], or a double antibody tyramide fluorescent immunostaining method, as described previously [4, 21]. Antibodies used are listed in Table 1.

**Table 1:** Details of immunohistochemical staining used in these studies.

Cell Type	Primary Ab	Fixative
Gonadotrophs	Guinea Pig anti-FSHβ (NIDDK-NIH)	Bouins
Gonadotrophs	Mouse anti-LHβ (Gift from Dr. W. Colin Duncan)	10% NBF
Thyrotrophs	Guinea Pig anti-TSHβ (AFP3035990P, NIDDK-NIH), gift from Prof. Alan McNeilly	Bouins
Corticotrophs	Mouse anti-ACTH (#M3501, DAKO)	Bouins
Somatotrophs	Rabbit anti-GH (A0570, DAKO)	Bouins
Lactotrophs	Rabbit anti- PRL (A0569, DAKO)	Bouins
AR^+ve^ Cells	Rabbit anti-AR (ab74272, Abcam)	Bouins or 10% NBF
ERα^+ve^ cells	Rabbit anti-ERα (MC-20) (Santa Cruz #sc-542)	Bouins
YFP/GFP	Rabbit anti-YFP/GFP (Life Technologies A-11122)	Bouins

### Quantification of the proportion of endocrine cells expressing AR or ESR1 in the anterior pituitary

Double immunofluorescent labelling of cell-specific pituitary hormones and either AR or ESR1 was performed on tissue sections cut from the middle of the pituitary of C57Bl6/J mice (n = 3). Three images per section were captured using a Zeiss LSM 510 confocal microscope coupled with LSM software (Carl Zeiss Ltd, Welwyn Garden City, UK) and used to determine colocalisation of AR or ESR1 with pituitary hormone products. Counting was done in Adobe Photoshop. Only cells with ≥ 50% cytoplasmic staining for hormones were counted. Counts were expressed as the percentage of hormone positive cells that were also AR/ESR1 positive.

### Determination of the relative volume of endocrine cells comprising the anterior pituitary

Three longitudinal sections per pituitary (approximately 25, 50 and 75% of the way through) from control (n = 5) and Foxg1^Cre/+^; AR^fl/y^ (n = 5) animals were analysed using a Zeiss Imager A1 microscope (Carl Zeiss Ltd, Welwyn Garden City, UK) coupled with a Qimaging QICAM Fast 1394 digital camera (Qimaging, Surrey, BC, Canada) and a Prior ProScan automated stage (Prior Scientific Instrument Ltd, Cambridge, UK) driven by Image-Pro Plus 7.0 software (MediaCybernetics, Rockville, MD, USA). Using the software’s stereologer plug-in, a tiled-image of the entire pituitary section was generated, from which an area of interest (AOI; the anterior lobe) was selected. The software then generated 50 random fields within the selected AOI. The automatic stage was then driven between fields, placing a counting grid over the tissue. Points falling over hormone-positive cells were counted. Between 15 and 20 fields were typically counted to ensure a standard error (SE) for counting of <5%: SE. Only fields that were occupied by ≥50% anterior lobe were scored. The proportion of the tissue occupied by hormone positive cells (the relative volume, RV) was then calculated as RV = (Ppi/Pt) x100 (Ppi = number of points over hormone positive cells expressed as a percentage of the total number of points counted. Pt = Total number of points counted).

### Preparation of pituitary cDNA

RNA was isolated from frozen pituitaries from n = 8 d80 Foxg1^Cre/+^; AR^fl/y^ and control mice using the RNeasy Mini extraction kit with RNase-free DNase on-the-column digestion kit (Qiagen, Crawley, UK) according to the manufacturer’s instructions. RNA was quantified using a NanoDrop 1000 spectrophotometer (Thermo Fisher Scientific, Waltham, MA, USA). Random hexamer primed cDNA was prepared using the SuperScript VILO cDNA synthesis kit (Life Technologies) according to manufacturer’s instructions.

### Determination of deletion of AR exon 2

Evaluation of exon 2 ablation in AR mRNA was based on previously published protocols [22]. RT-PCR was performed on pituitary cDNA using BioMix Red Taq polymerase (Bioline, London, UK) and primers located in AR exons 1 and 3 (forward AAGCAGGTAGCTCTGGGACA; reverse CGTTTCTGCTGGCACATAGA). PCR amplification products were resolved using a QiaXcel capillary system (Qiagen, UK) with an amplicon of 765 bp if AR exon 2 was present and 613 bp if exon 2 had been ablated by Cre recombinase.

### Quantitative RT-PCR (qPCR)

Multiplex qPCR was performed on pituitary cDNA for the genes listed in Table 2 using an ABI Prism 7900 Sequence Detection System (Applied Biosystems) and the Roche Universal Probe library (Roche, Welwyn, UK). Due to its high expression level, the expression of *Prl* was related to an internal housekeeping gene assay for 18s rRNA (Life Technologies) whereas the expression of all other genes were related to an internal housekeeping gene assay for *Actb* (Roche, Welwyn, UK) as described previously [23]. Resulting data were analysed using the ΔΔCt method.

**Table 2:** Primers and Roche UPL probes for qRT-PCR assays used in these studies.

Gene	Forward primer	Reverse primer	Probe
***Cga***	aaatatgcagctgtcattctgg	cctttagtttacattctgggcaac	56
***Fshb***	agacagctgactgcacagga	tcacagctatggcagcagat	67
***Lhb***	ctcagccagtgtgcacctac	ggaaaggagactatggggtctac	71
***Tshb***	aagagctggggttgttcaaa	acaagcaagagcaaaaagcac	101
***Pomc***	ggcctttcccctagagttca	gacctgctccaagcctaatg	11
***Gh***	ggaaaagcactagcctcctg	gcttggcaatggctacaga	13
***Prl***	tgttcccagcagtcaccat	cagcaacaggaggagtgtcc	26
***Gnrhr***	ggcatcaggccttctacaac	tggactgattcagctgtagtttg	34
***Esr1***	tgcaatgactatgcctctgg	ttgtagctggacacatgtagtcatt	93
***Gr***	caaagattgcaggtatcctatgaa	cttggctcttcagaccttcc	91
***Cyp19a1***	ccactcctgctgatcatgg	tcccagacagtagccaggac	2

### Quantification of plasma hormone levels

Immediately after culling, blood was collected from n≥8 control and Foxg1^Cre/+^; AR^fl/y^ mice by cardiac puncture with a syringe and needle pre-treated with heparin. Plasma was separated by centrifugation and stored at-80. Testosterone was measured using a radioimmunoassay as previously described [24]. LH and FSH were measured using in-house designed ELISAs as previously described [25]. Commercially available ELISAs for prolactin (Abcam #ab100736), GH (Merck Millipore EZRMGH-45K), ACTH (Antibodies Online #ABIN415571) and TSH (Antibodies Online #ABIN415519) were performed according to manufacturer’s instructions. All samples were run in duplicate in a single assay for each hormone.

### Western blotting

Western blotting for ESR1 was performed as reported previously [26] on d100 control (n = 4) and Foxg1^Cre/+^; AR^fl/y^ (n = 4) pituitaries. Blots were probed with primary antibodies ESR1 (Santa Cruz sc-542) and tyrosinated TUBA (α-tubulin) isoforms (Abcam ab6160). Primary antibodies were detected using donkey anti-rabbit IRDye 680RD and goat anti-rat IRDye 800CW (LI-COR Biosciences). Detection was carried out using the LI-COR Odyssey imaging system (LI-COR biosciences) according to manufacturer’s instructions.

### Statistical analysis

Data were analysed using GraphPad Prism (version 5; GraphPad Software Inc., San Diego, CA, USA) using a two-tailed unpaired t test with Welch’s correction (if comparing two groups) or a one-way ANOVA with Bonferroni post-hoc test (if comparing more than two groups). Values are expressed as means ± S.E.M. Values are stated as ‘increased’ or ‘decreased’ compared to controls only if they are statistically significantly different.

## Results

### Foxg1-Cre ablates AR from the pituitary gland but circulating testosterone levels are not affected

**Fig 1:**
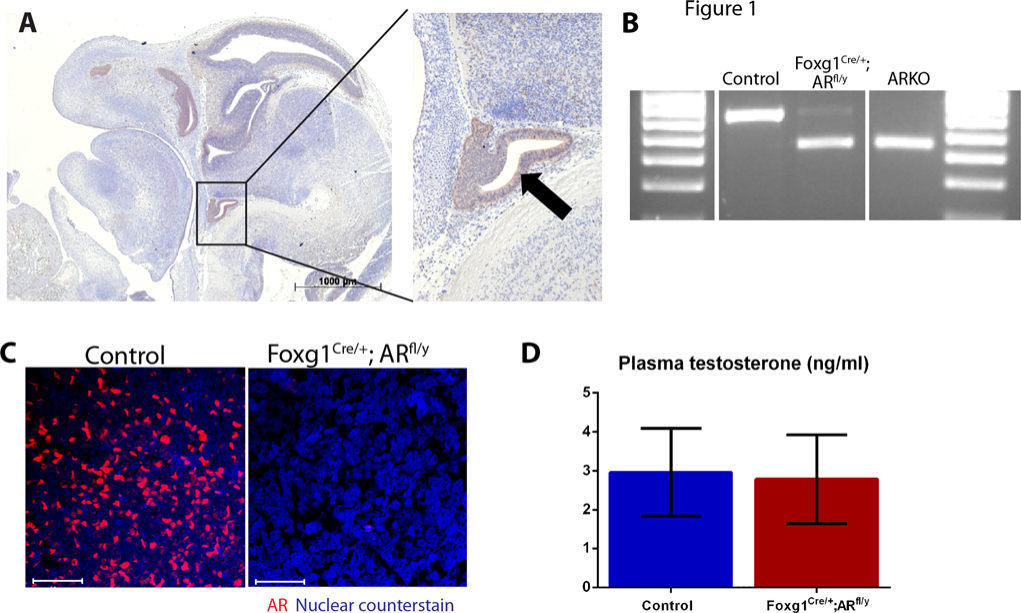
Foxg1-Cre recombines genomic *Ar* and results in loss of AR protein in Foxg1^Cre/+^; AR^fl/y^ pituitaries. (A) YFP staining can be seen in the Rathke’s pouch (arrow and magnification) of Foxg1-YFP embryos at e12.5 (B) When analysed by PCR, genomic DNA of pituitaries of d2 control mice showed unrecombined *Ar* (upper band, 765 bp), but nearly all genomic *Ar* present in pituitaries of d2 Foxg1^Cre/+^; AR^fl/y^ mice has been recombined by Cre recombinase (lower band, 613) as seen in the complete ARKO (C) Adult control mice show AR immunostaining (red) in the pituitary, but this is completely lost in Foxg1^Cre/+^; AR^fl/y^ mice. Nuclear counterstain is blue. Scale bars are 50μm. (D) No significant difference was seen between Foxg1^Cre/+^; AR^fl/y^ and control plasma levels of testosterone.

Breeding of R26R-EYFP female mice [27] to Foxg1-Cre males, results in Foxg1-YFP offspring where any permanent Cre-mediated recombination event induces irreversible YFP expression in that cell and its daughters. Immunohistochemical localisation of YFP in e12.5 embryos revealed expression throughout Rathke’s pouch (the anlage of the pituitary) (Fig. 1A). Previous studies have demonstrated that Foxg1 is not expressed in the hypothalamus [19], as such, we concluded that the Foxg1-Cre has utility for targeting of AR in the male pituitary. To generate Foxg1^Cre/+^; AR^fl/y^ mice, hemizygous Foxg1-Cre males are bred to homozygous AR^fl/fl^ females [20]. When analysed by PCR, genomic DNA of pituitaries of day (d) 2 control mice show unrecombined AR, but almost all genomic DNA present in pituitaries of d2 Foxg1^Cre/+^; AR^fl/y^ mice is recombined by Cre recombinase to generate a null allele of AR (Fig. 1B). Adult control mice show AR immunostaining in the pituitary, but this is completely absent in pituitaries of Foxg1^Cre/+^; AR^fl/y^ mice (Fig. 1C) demonstrating that AR ablation from fetal life is permanent, completely abolishing any subsequent action of AR in the developing and adult pituitary. Surprisingly, ablation of pituitary AR does not result in any changes to circulating testosterone level (Fig. 1D), questioning the current paradigm of testosterone-mediated negative feedback within the HPG axis.

### AR is expressed in all anterior pituitary cell types

**Fig 2:**
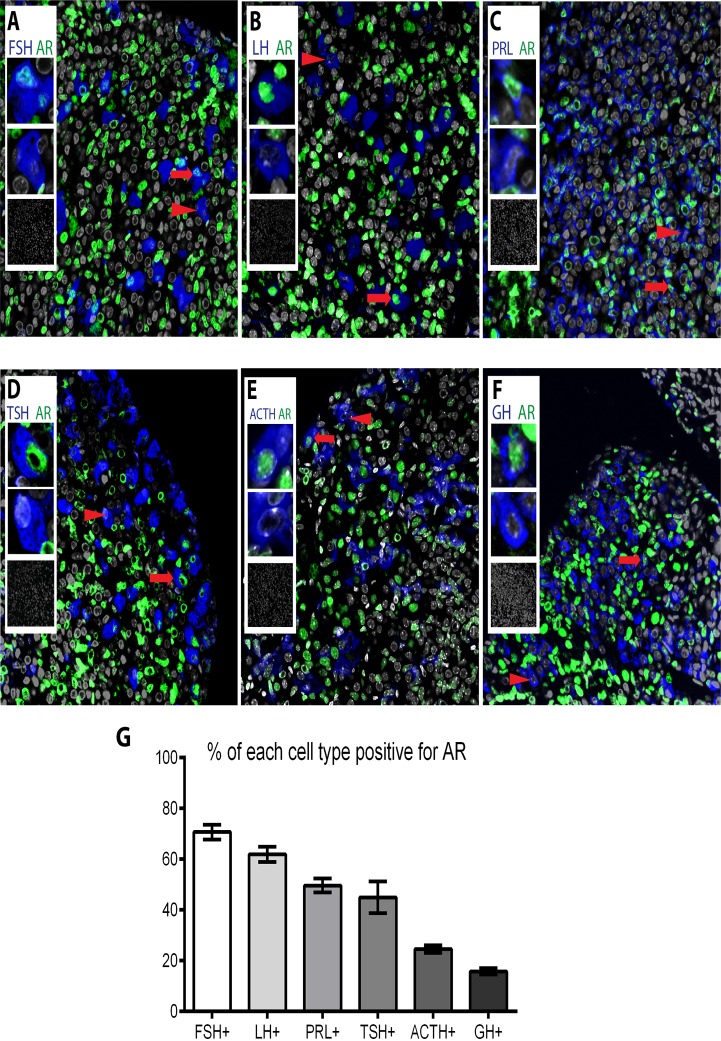
All pituitary endocrine cell populations express AR at different percentages. Subpopulations of (A) FSH-positive gonadotrophs (blue), (B) LH-positive gonadotrophs (blue), (C) PRL-positive lactotrophs (blue), (D) TSH-positive thyrotrophs (E) ACTH-positive corticotrophs (F) GH-positive somatotrophs stain positive for AR (green). Arrows point to AR positive cells and arrowheads to AR negative cells, insets show magnifications of AR positive and negative cells, and no-primary controls. (G) When quantified, the percentage of each endocrine cell population positive for AR was 70.7% ±2.9 for FSH-positive gonadotrophs, 61.9% ±3.0 for LH-positive gonadotrophs, 49.65% ±2.78 for PRL-positive lactotrophs, 44.97% ±6.28 for TSH-positive thyrotrophs, 24.56% ±1.52 for ACTH-positive corticotrophs and 15.77% ±1.27 for GH-positive somatotrophs.

Prior to investigating the impact of AR ablation, double-immunofluorescence experiments were performed on pituitaries from wild-type C57BL/6J mice to determine which endocrine cells express AR. Localisation of cell-specific hormones and AR protein on the same tissue section revealed all endocrine cell-types of the male anterior pituitary gland express AR (Fig. 2A-G). The population with the highest percentage of cells expressing AR is FSH-positive gonadotrophs (70.7% ±2.9) followed by LH-positive gonadotrophs (61.9% ±3.0), PRL-positive lactotrophs (49.65% ±2.78), TSH-positive thyrotrophs (44.97% ±6.28), ACTH-positive corticotrophs (24.56% ±1.52) and the lowest percentage of AR staining in GH-positive somatotrophs (15.77% ±1.27).

### Pituitary cell type volume does not change in Foxg1^Cre/+^; AR^fl/y^ mice

**Fig 3:**
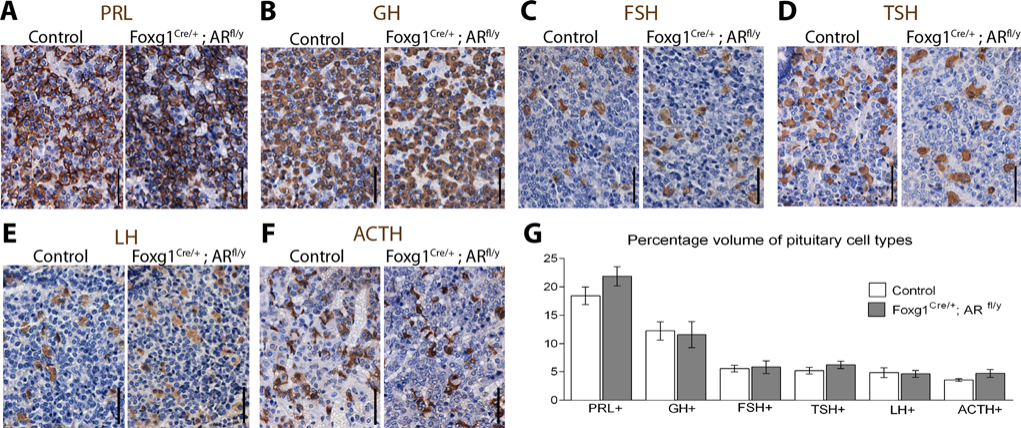
Cell population volume does not change in Foxg1^Cre/+^; AR^fl/y^ pituitaries. Subpopulations of pituitary cells stain positive for (A) PRL, (B) GH, (C) FSH, (D) TSH (E) LH (F) ACTH. (G) When volume is quantified there is no significant difference between control and Foxg1^Cre/+^; AR^fl/y^ for PRL-positive lactotrophs (Control 18.4% ±1.6, Foxg1^Cre/+^; AR^fl/y^ 21.9% ±1.7), GH-positive somatotrophs (Control 12.3% ±1.6, Foxg1^Cre/+^; AR^fl/y^ 11.6% ±2.3), FSH-positive gonadotrophs (Control 5.6% ±0.6, Foxg1^Cre/+^; AR^fl/y^ 5.8% ±1.1), TSH-positive thyrotrophs (Control 5.2% ±0.6, Foxg1^Cre/+^; AR^fl/y^ 6.2% ±0.7), LH-positive gonadotrophs (Control 4.8% ±0.8, Foxg1^Cre/+^; AR^fl/y^ 4.6% ±0.6) and ACTH-positive corticotrophs (Control 3.6% ±0.2, Foxg1^Cre/+^; AR^fl/y^ 4.7% ±0.7).

Androgens acting *via* AR have been previously shown to have important roles in the programming of multiple tissues in the developing male embryo. Therefore, any developmental consequence of insufficient androgen-AR signalling during embryonic development on final percentage cell volume of the different populations of endocrine cells in the anterior pituitary of adult Foxg1^Cre/+^; AR^fl/y^ males was assessed. No gross differences between the pituitary morphology or size of the two groups is noted during dissection. No differences in histological morphology are noted between control and Foxg1^Cre/+^; AR^fl/y^ pituitaries. Immunohistochemistry was performed to identify each of the pituitary cell types (Fig. 3A-F) and cell population volumes were quantified as a percentage of the anterior pituitary total cell volume. No significant difference in the volume of the anterior pituitary occupied by the specific endocrine cell populations is observed for PRL-positive lactotrophs (control 18.4% ±1.6, Foxg1^Cre/+^; AR^fl/y^ 21.9% ±1.7), GH-positive somatotrophs (control 12.3% ±1.6, Foxg1^Cre/+^; AR^fl/y^ 11.6% ±2.3), FSH-positive gonadotrophs (control 5.6% ±0.6, Foxg1^Cre/+^; AR^fl/y^ 5.8% ±1.1), TSH-positive thyrotrophs (control 5.2% ±0.6, Foxg1^Cre/+^; AR^fl/y^ 6.2% ±0.7), LH-positive gonadotrophs (control 4.8% ±0.8, Foxg1^Cre/+^; AR^fl/y^ 4.6% ±0.6), ACTH-positive corticotrophs (control 3.6% ±0.2, Foxg1^Cre/+^; AR^fl/y^ 4.7% ±0.7) when measured by stereology (Fig. 3G). Together these data suggest pituitary AR-signalling is dispensable for the attainment of normal cellular content in the adult male pituitary.

### Circulating gonadotrophin levels are unchanged in Foxg1^Cre/+^; AR^fl/y^ mice

**Fig 4:**
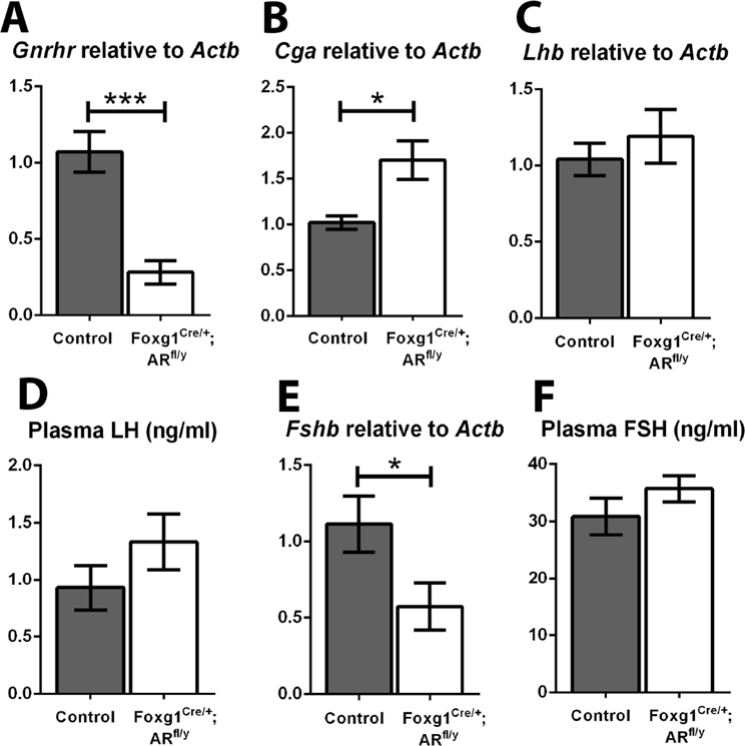
No changes are seen in gonadotrophin mRNAs or circulating hormone levels in Foxg1^Cre/+^; AR^fl/y^ mice. (A) There is a significant decrease in transcript of gonadotrophin releasing hormone receptor (*Gnrhr*, p<0.001) in the Foxg1^Cre/+^; AR^fl/y^ pituitary compared to controls. (B) There is a significant increase in the normalised pituitary transcript for common glycoprotein subunit-α (*Cga*, p<0.05) in Foxg1^Cre/+^; AR^fl/y^ s. (C) There is no significant difference in luteinising hormone-β transcript (*Lhb*) between controls and Foxg1^Cre/+^; AR^fl/y^ s. (D) No significant difference was seen between Foxg1^Cre/+^; AR^fl/y^ and control plasma levels of LH (Control 0.93 ±0.20, Foxg1^Cre/+^; AR^fl/y^ 1.33 ±0.24 ng/ml), (E) There is a significant decrease in the Foxg1^Cre/+^; AR^fl/y^ pituitary of follicle stimulating hormone-β transcript (*Fshb*, p<0.05). (F) No significant difference was seen between Foxg1^Cre/+^; AR^fl/y^ and control plasma levels of FSH (Control 30.9 ±3.19, Foxg1^Cre/+^; AR^fl/y^ 35.72 ±2.31 ng/ml).

To empirically determine whether pituitary androgen receptor is required for the control of gonadotrophin production and release, expression levels of transcripts encoding pituitary *Gnrhr* (the receptor for GnRH) and gonadotrophin subunits, and concentrations of plasma gonadotrophins were compared between control and Foxg1^Cre/+^; AR^fl/y^ mice. *Gnrhr* transcript levels are significantly reduced (p<0.001) suggesting loss of AR may have altered the sensitivity of gonadotrophs to GnRH (Fig. 4A). A significant increase in common glycoprotein subunit-α (*Cga*, Fig. 4B) is also seen in Foxg1^Cre/+^; AR^fl/y^ pituitaries compared to controls, whilst no change is observed in luteinising hormone-β transcript (*Lhb*, Fig. 4C). Surprisingly, despite reductions in both *Gnrhr* and *Cga* transcription, we observe no change in plasma LH concentrations compared to littermate controls (control 0.93 ±0.20, Foxg1^Cre/+^; AR^fl/y^ 1.33 ±0.24 ng/ml, Fig. 4D). Follicle stimulating hormone-β (*Fshb*) transcript is significantly decreased in Foxg1^Cre/+^; AR^fl/y^ males compared to controls (Fig. 4E) but again, this i not reflected in a change in the level of plasma FSH (control 30.9 ±3.19, Foxg1^Cre/+^; AR^fl/y^ 35.72 ±2.31 ng/ml), Fig. 4E). Together these data demonstrate that pituitary AR is dispensable for regulation of circulating gonadotrophin concentrations in the male mouse.

**Fig 5:**
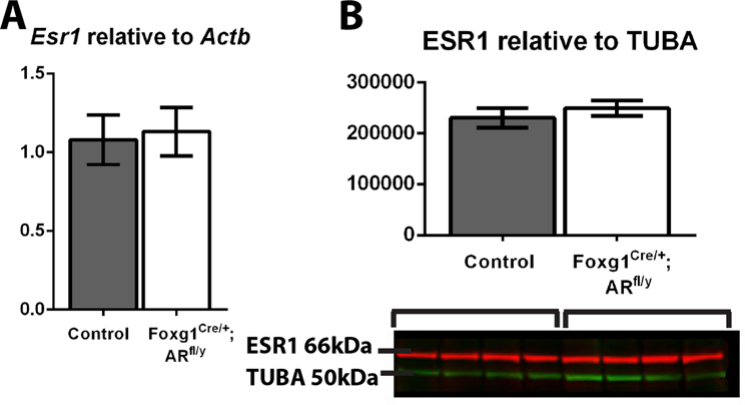
ESR1 transcript and protein levels do not change in Foxg1^Cre/+^; AR^fl/y^ pituitaries. (A) No significant difference was seen in *Esr1* transcript levels or (B) ESR1 protein levels (normalised to TUBA) in Foxg1^Cre/+^; AR^fl/y^ pituitaries compared to controls.

Estrogen signalling is thought to be responsible for a proportion of the feedback attributed to testosterone at the hypothalamus and pituitary, and estrogen receptor α (ESR1) levels are shown to increase in some tissues when AR is ablated or downregulated [28] [29] [16], raising the possibility that increased or retained estrogen signalling could explain the lack of impact loss of AR has on circulating gonadotrophin levels. However, no significant difference is seen in *Esr1* transcript levels (Fig. 5A) or ESR1 protein levels (Fig. 5B) in Foxg1^Cre/+^; AR^fl/y^ pituitaries compared to controls, and expression of *Cyp19a1* (aromatase) cannot be detected in either control or Foxg1^Cre/+^; AR^fl/y^ male pituitaries (data not shown), suggesting compensation by modulation of estrogen signalling does not underpin any theoretical ‘rescue’ of circulating gonadotrophin levels.

### Ablation of pituitary AR does not affect some pituitary products

**Fig 6:**
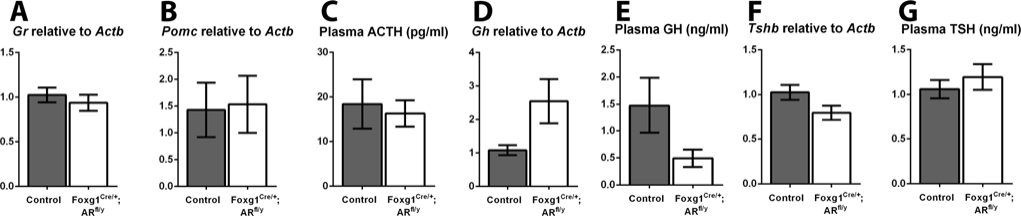
No changes are seen in some pituitary hormone mRNAs or circulating products in Foxg1^Cre/+^; AR^fl/y^ mice. (A) There is no significant difference in transcript of glucocorticoid receptor (*Gr*) between Foxg1^Cre/+^; AR^fl/y^ and control pituitaries. (B) There is no significant difference in transcript of pro-opiomelanocortin (*Pomc*) between Foxg1^Cre/+^; AR^fl/y^ and control pituitaries. (C) No significant difference was seen between Foxg1^Cre/+^; AR^fl/y^ and control plasma levels of ACTH (Control 18.42 ±5.52, Foxg1^Cre/+^; AR^fl/y^ 16.30 ±2.97 pg/ml). (D) There is no significant difference in transcript of growth hormone (*Gh*), between Foxg1^Cre/+^; AR^fl/y^ and control pituitaries. (E) No significant difference was seen between Foxg1^Cre/+^; AR^fl/y^ and control plasma levels of GH (Control 1.48 ±0.51, Foxg1^Cre/+^; AR^fl/y^ 0.50 ±0.16 ng/ml). (F) There is no significant difference in transcript of thyroid-stimulating hormone-β (*Tsh*) between Foxg1^Cre/+^; AR^fl/y^ and control pituitaries. (G) No significant difference was seen between Foxg1^Cre/+^; AR^fl/y^ and control plasma levels of TSH (Control 1.06 ±0.11, Foxg1^Cre/+^; AR^fl/y^ 1.20 ±0.14 ng/ml).

Having demonstrated AR is dispensable for regulation of circulating gonadotrophin levels, we next asked whether loss of AR impacted other pituitary products. Since previously published literature has suggested AR plays a role in the regulation of pituitary glucocorticoid receptor (GR) expression [4], we quantified *Gr* transcript levels, but no difference is noted between Foxg1^Cre/+^; AR^fl/y^ and control animals (Fig. 6A), suggesting pituitary AR is not a major regulator of pituitary GR expression. Since all pituitary endocrine cells express AR (Fig. 1), the expression levels of transcripts encoding other pituitary hormone subunits and concentrations of plasma pituitary products were compared between control and Foxg1^Cre/+^; AR^fl/y^ mice. No difference is observed in either the transcript for pro-opiomelanocortin (*Pomc* the precursor transcript of ACTH, Fig. 6B) or its circulating form ACTH (control 18.42 ±5.52, Foxg1^Cre/+^; AR^fl/y^ 16.30 ±2.97 pg/ml Fig. 6C). Likewise, no difference is observed in either the transcript for growth hormone (*Gh*, Fig. 6D) or circulating GH (control 1.48 ±0.51, Foxg1^Cre/+^; AR^fl/y^ 0.50 ±0.16 ng/ml, Fig. 6E). No change is seen in thyroid-stimulating hormone-β (*Tsh*, Fig. 6F) or circulating TSH (control 1.06 ±0.11, Foxg1^Cre/+^; AR^fl/y^ 1.20 ±0.14 ng/ml, Fig. 6G).

### Pituitary AR functions to suppress prolactin production by the male pituitary

**Fig 7:**
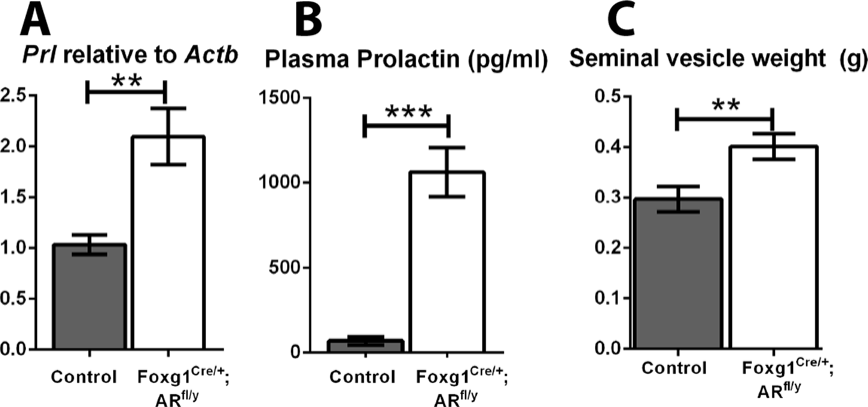
Foxg1^Cre/+^; AR^fl/y^ mice have an increase in circulating prolactin and heavier seminal vesicles. (A) There is a significant increase of prolactin transcript (*Prl*, p<0.01) in the Foxg1^Cre/+^; AR^fl/y^ pituitary compared to control. (B) Plasma prolactin levels were significantly increased in Foxg1^Cre/+^; AR^fl/y^ mice (1062 ±144.7 pg/ml) compared to controls (69.0 ±24.3 pg/ml, p<0.001). (C) Adult Foxg1^Cre/+^; AR^fl/y^ mice have a significant increase in seminal vesicle weight (p<0.01).

Surprisingly, a significant increase in pituitary prolactin transcript (*Prl*, Fig. 7A) and a 15-fold increase in plasma prolactin levels (Control 69.0 ±24.3, Foxg1^Cre/+^; AR^fl/y^ 1062 ±144.7 pg/ml, Fig. 7B) are observed in Foxg1^Cre/+^; AR^fl/y^ mice compared to controls. Furthermore, seminal vesicle weight is significantly increased in Foxg1^Cre/+^; AR^fl/y^ mice (Fig. 7C), consistent with other rodent models of hyperprolactinaemia [30, 31].

### The percentage of lactotrophs expressing ESR1 does not change in Foxg1^Cre/+^; AR^fl/y^ mice

**Fig 8:**
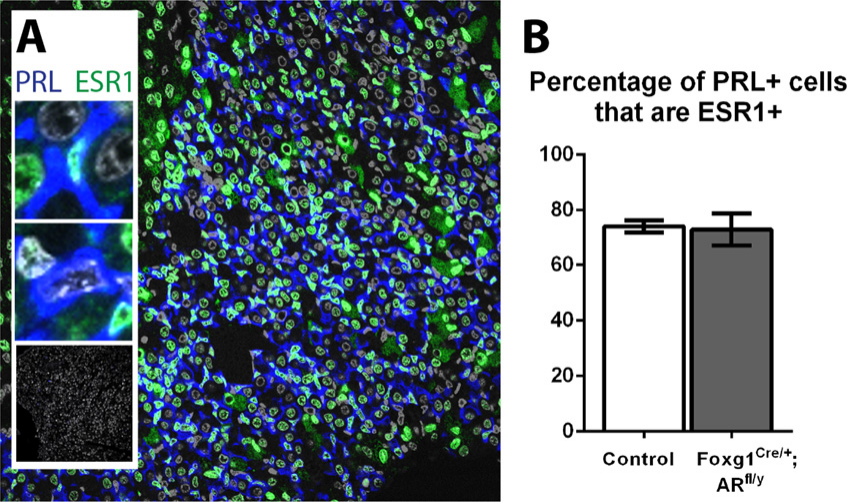
The percentage of lactotrophs expressing ESR1 does not change in Foxg1^Cre/+^; AR^fl/y^ mice. (A) A subpopulation of PRL-positive lactotrophs (blue) stain positive for ESR1 (green). Insets show magnifications of ESR1 positive and negative cells, and no-primary controls. (B) No significant difference was found in the percentage of lactotrophs that express ESR1 (Control 74.2% ±2.2, Foxg1^Cre/+^; AR^fl/y^ 73.1% ± 5.8).

Since estradiol is the main stimulator of lactotroph prolactin production [32], we next sought to determine whether ESR1 expression specifically in lactotrophs is altered in Foxg1^Cre/+^; AR^fl/y^ males after embryonic AR ablation. Double immunohistochemistry was performed for ESR1 and PRL (Fig. 8A) however, no significant difference is found in the percentage of lactotrophs that express ESR1 (Control 74.2%, Foxg1^Cre/+^; AR^fl/y^ 73.1%, Fig. 8B).

## Discussion

Production of testosterone by testicular Leydig cells is tightly regulated by the hypothalamic-pituitary-gonadal (HPG) axis, forming a homeostatic negative feedback loop, but exactly how this is affected is not entirely understood. In this study we examined a mouse line with ablation of androgen receptor in the pituitary (Foxg1^Cre/+^; AR^fl/y^) to empirically determine the role of pituitary androgen receptor in the control of male pituitary hormone production. Foxg1-Cre was active from fetal life and ablated androgen receptor in the pituitary. Surprisingly this resulted in no change in circulating testosterone levels, questioning the currently accepted paradigm of testosterone-mediated feedback at the level of the pituitary. Further to this observation, in this study we demonstrate that circulating gonadotrophin levels are unaffected by loss of pituitary AR, suggesting androgens do not mediate negative feedback on the HPG axis at the level of the pituitary in males. Instead, we show that pituitary AR is required for repression of prolactin production and release by the male pituitary.

The majority of Cre Recombinase mouse lines target multiple tissues; as such empirical establishment of sites of targeting is an essential step in validating the utility of any given line as fit for purpose [33]. Foxg1-Cre is active in the developing brain in the telencephalon, anterior optic vesicle, otic vesicle, facial and head ectoderm, olfactory epithelium, mid–hindbrain junction, and pharyngeal pouches [19]. Whilst these structures do not contribute towards the hypothalamus, Foxg1-Cre is active in Rathke’s pouch (the anlage of the pituitary) from e10 and previous studies use Foxg1-Cre to ablate a floxed gene of interest in the pituitary but not the hypothalamus [17, 18]. In addition, Foxg1-Cre is also expressed ectopically in some tissues outside of the brain, although the use of mice with 129SvJ and C57BL/6 backgrounds (as in the study) is shown to limit this [19]. We have ourselves previously used Foxg1-Cre to ablate androgen receptor action from the caput epididymal epithelium (CEARKO) [16]. In this case, the epididymal effects we observe were independently confirmed in a second model that did not have pituitary AR ablation [34], suggesting a localised impact of AR ablation at this distal site, with no impact on the central HPG axis. Given this, and the observation that Foxg1-Cre does not target the hypothalamus or the gonad, but only targets the pituitary component of the HPG axis, we conclude that, in addition to its utility for investigating epididymal AR function, the Foxg1^Cre/+^; AR^fl/y^ mouse model also provides a unique opportunity to determine the role of AR-signalling in the male pituitary.

### Role of steroid hormone receptors in gonadotrophin release

The male HPG axis paradigm is centred on testosterone providing a negative feedback repression at both the hypothalamus (GnRH production) and pituitary (LH production). Our data show no change in circulating LH or testosterone [16] levels when AR is selectively genetically ablated from the male mouse pituitary. One explanation is that AR signalling, though dispensable at the pituitary level is required at the hypothalamic level. Since GnRH-producing neurons do not express AR [35] the level of hypothalamic repression must be upstream of GnRH production, potentially at the level of kisspeptin-producing neurons, which do express AR [36]. If AR is indeed dispensable at the pituitary level for control of the gonadotrophins, the possibility exists that gonadal steroid feedback is through aromatisation of androgen to estrogen and subsequent signalling through ERα. However, the evidence for this is conflicting with some studies reporting that circulating LH levels are unaltered in global ERα knockout mice, [37, 38] while others report an increase in circulating LH [39]. Some studies suggest that only exogenous androgens can supress the increase in circulating LH levels following castration of male mice [39], others suggest estrogens are required [37]. The phenotype of global ERα knockout mice is complicated by ablation in the efferent ducts that renders sperm non-functional post epididymal transit, so this model cannot be used to define if HPG effects contribute towards their infertility. Two models of pituitary-specific ERKO males have been noted to be ‘fertile’ [40, 41], although no further characterisation of their reproductive parameters was undertaken. Global ERβ knockout mice do not appear to have defects in their HPG axis [42], so it is unlikely that estrogen signalling in the male hypothalamic-pituitary unit is *via* ERβ. Even if normal pituitary feedback is maintained by ERα in the absence of AR, our data show that there is no compensatory upregulation of ERα mRNA or protein in the Foxg1^Cre/+^; AR^fl/y^. We can also not identify aromatase transcript in the control or Foxg1^Cre/+^; AR^fl/y^ male mouse pituitary, suggesting that the synthesis of estrogen that may be involved in the pituitary feedback is at a distal site. This is contrary to data in male rats [43] which do express aromatase.

Interestingly, levels of gonadotrophin-releasing hormone receptor (Gnrhr) transcript are significantly reduced in the Foxg1^Cre/+^; AR^fl/y^ compared to controls. This could potentially be a site of androgen feedback by decreasing the number of pituitary receptors for GnRHR to bind and stimulate a response. However, this is contrary to published literature which shows that castration has no effect on pituitary GnRHR levels in the mouse [44], and despite this reduction in *Gnrhr*, we show that circulating gonadotrophin levels do not change. Interestingly, a recently published model of specific androgen receptor ablation in gonadotrophs in female mice also showed that basal LH level is not changed, although pre-ovulatory LH surge is decreased [45]. Basal and surge FSH levels in these females are also decreased, indicating that the role of AR in gonadotrophs may be sexually dimorphic.

### Role of AR in other pituitary cell types

The HPG axis paradigm focuses on androgen receptor feedback to pituitary gonadotrophs. Despite this, our results show that a sub-population of all other pituitary endocrine cell types express androgen receptor, implying that androgen signalling is involved in their function. A role for pituitary AR in the regulation of pituitary glucocorticoid receptor expression has been postulated by Miyamoto *et al* [4]. The authors set out to explore the molecular mechanism behind late-onset obesity in androgen receptor knock-out (ARKO) mice, which they suggest is caused by a hyper-corticoid state, driven by high levels of pituitary-derived ACTH as a consequence of impairment in the hypothalamic-pituitary-adrenal (HPA) axis. Using a luciferase reporter assay in a pituitary cell culture system, the authors suggest that GR expression is under positive control by the AR, and they arrive at the conclusion that AR functions in the negative-feedback regulation of glucocorticoid production by up-regulating GR expression in the pituitary. However, these conclusions are not supported by data from the Foxg1^Cre/+^; AR^fl/y^ mouse. There are two possibilities to explain this discrepancy. Firstly, the mouse model used has a total ablation of AR, which extends the possibility that the phenotype is a result of AR ablation in another cell/tissue type. Second, the use of a cell culture system does not take into account the behaviour of pituitary cells as a network *in vivo*.

The hypothalamic-pituitary unit is sexually dimorphic and structural and functional differences between males and females are potentiated by steroid exposure both neonatally and at puberty. An example of neonatal steroids affecting development is the oxytocin pulse generator that is neonatally inactivated in males as testicular testosterone is aromatised into estrogen and acts *via* ERα to cause the neurons to undergo apoptosis. Females produce no gonadal steroids at this age so the neurons persist [46]. The cellular composition of the anterior pituitary does not differ between the sexes until after pubertal onset when females begin to develop larger anterior pituitaries with a higher percentage of lactotrophs, while pituitaries of males contain a higher percentage of somatotrophs [47]. This dimorphism is due to differences in both the neonatal and postpubertal steroid milieu. In Foxg1^Cre/+^; AR^fl/y^ mice there is no change in somatotroph volume, and no significant difference in circulating GH levels, implying that pituitary androgen receptor is not involved in the development of this dimorphism.

The most striking result identified in the Foxg1^Cre/+^; AR^fl/y^ mouse was the increase in both pituitary transcript and serum hormone prolactin levels, which validates a previously published postulation of androgen repression of prolactin release [48]. Control of serum prolactin levels is *via* a balance between stimulation by estrogen and inhibition by dopamine produced by the tuberoinfundibular dopaminergic neurones of the hypothalamus [32, 49]. Estrogen and dopamine antagonise each other to regulate lactotroph endocrine functions including the secretion and synthesis of prolactin, lactotroph proliferation and gene expression. DHT has previously been shown to reverse the stimulatory effect of estradiol on *Prl* mRNA levels in rats [50], which supports our observation of prolactin mRNA and protein increase when androgen-signalling repression is removed. It is yet to be determined if this repression is directly through androgen receptor binding to the prolactin promoter or through an indirect mechanism in adulthood.

Male rats have been observed to have fewer lactotrophs than females [47]. Neonatal orchidectomy of males resulted in an increase of lactotrophs, but adult orchidectomy did not change this number, implying that prepubertal gonadal steroid hormones are involved in regulation of lactotroph specification. In our model of pituitary androgen receptor ablation, we do not see a change in relative cell volume despite the change in serum prolactin so there is unlikely to be a developmental programming effect on lactotroph number potentiated by AR. This may be because signalling *via* estrogen is instead responsible for the number of lactotrophs and orchidectomy of rats removes both androgens and androgens as the source of estrogen through aromatisation.

Despite its well-characterised role in lactogenesis in female mammals, the role of prolactin in males is ill defined. Prolactin treatment has been shown to increase testosterone secretion and endogenous prolactin is required for the complete expression of the stimulatory action of LH on T secretion in adult male rats [51]. Both prolactin [52] and prolactin receptor [53] knock-out male mice are fertile suggesting that prolactin does not have any vital effects on male fertility, but prolactin knock-out males display reduced LH levels and weights of seminal vesicles and ventral prostate suggesting it may have a trophic effect on these organs. Clinical cases of hyperprolactinaemia associated with prolactinomas report low testosterone, decreased libido and erectile dysfunction [54] as well as low sperm count [55]. Mice with induced hyperprolactinaemia show increased seminal vesicle weight [30, 31], which is also seen in the Foxg1^Cre/+^; AR^fl/y^.

Despite AR expression being present in all pituitary cell types, there was limited effect of AR ablation in most cell types other than the striking effect on prolactin secretion. Volume of pituitary cell types as a percentage of the anterior lobe does not change; there are also no gross differences between morphology and size of pituitaries of control and Foxg1^Cre/+^; AR^fl/y^ mice. Ablation of AR from the embryonic pituitary gland (as occurs in this model) means that the Foxg1^Cre/+^; AR^fl/y^ pituitary has developed without exposure to androgens (although any effects on development from aromatisation to estrogen would still have occurred). Because of this it is difficult to elucidate whether any changes are due to a change in development of the pituitary or an acute effect of the lack of AR signalling. Addressing this would require a model of adult-onset genetic ablation of pituitary androgen receptor.

In conclusion, this study provides new insight into the regulation of pituitary endocrine hormones by androgens. It is a central paradigm of male reproductive biology that androgens act at both the hypothalamus and pituitary to repress the release of LH. However growing evidence (including that generated in this study) demonstrates that the feedback mechanism is independent of AR in the pituitary. The results we present here also strongly suggest a role of AR is to repress prolactin secretion in males, and that ablating AR from the pituitary removes this repression resulting in a novel model of hyperprolactinaemia.

## References

[1] Tilbrook AJ, Clarke IJ. Negative feedback regulation of the secretion and actions of gonadotropin-releasing hormone in males. Biology of reproduction. 2001;64(3): 735–42. 11207186 10.1095/biolreprod64.3.735

[2] Matsumoto AM. Effects of chronic testosterone administration in normal men: safety and efficacy of high dosage testosterone and parallel dose-dependent suppression of luteinizing hormone, follicle-stimulating hormone, and sperm production. The Journal of clinical endocrinology and metabolism. 1990;70(1): 282–7. 2104626 10.1210/jcem-70-1-282

[3] Migeon BR, Brown TR, Axelman J, Migeon CJ. Studies of the locus for androgen receptor: localization on the human X chromosome and evidence for homology with the Tfm locus in the mouse. Proceedings of the National Academy of Sciences of the United States of America. 1981;78(10): 6339–43. 6947233 10.1073/pnas.78.10.6339 PMC349034

[4] Miyamoto J, Matsumoto T, Shiina H, Inoue K, Takada I, Ito S, et al The pituitary function of androgen receptor constitutes a glucocorticoid production circuit. Molecular and cellular biology. 2007;27(13): 4807–14. 17470551 10.1128/MCB.02039-06 PMC1951475

[5] Gaspar ML, Meo T, Bourgarel P, Guenet JL, Tosi M. A single base deletion in the Tfm androgen receptor gene creates a short-lived messenger RNA that directs internal translation initiation. Proceedings of the National Academy of Sciences of the United States of America. 1991;88(19): 8606–10. 1924321 10.1073/pnas.88.19.8606 PMC52558

[6] Amador AG, Parkening TA, Beamer WG, Bartke A, Collins TJ. Testicular LH receptors and circulating hormone levels in three mouse models for inherited diseases (Tfm/y, lit/lit and hyt/hyt). Endocrinologia experimentalis. 1986;20(4): 349–58. 3100274

[7] Hughes IA, Davies JD, Bunch TI, Pasterski V, Mastroyannopoulou K, MacDougall J. Androgen insensitivity syndrome. Lancet. 2012;380(9851): 1419–28. 10.1016/S0140-6736(12)60071-3 22698698

[8] Pitteloud N, Dwyer AA, DeCruz S, Lee H, Boepple PA, Crowley WF Jr, et al Inhibition of luteinizing hormone secretion by testosterone in men requires aromatization for its pituitary but not its hypothalamic effects: evidence from the tandem study of normal and gonadotropin-releasing hormone-deficient men. The Journal of clinical endocrinology and metabolism. 2008;93(3): 784–91. 18073301 10.1210/jc.2007-2156 PMC2266963

[9] Hayes FJ, Seminara SB, Decruz S, Boepple PA, Crowley WF Jr. Aromatase inhibition in the human male reveals a hypothalamic site of estrogen feedback. The Journal of clinical endocrinology and metabolism. 2000;85(9): 3027–35. 10999781 10.1210/jcem.85.9.6795

[10] Bagatell CJ, Dahl KD, Bremner WJ. The direct pituitary effect of testosterone to inhibit gonadotropin secretion in men is partially mediated by aromatization to estradiol. Journal of andrology. 1994;15(1): 15–21. 8188534

[11] Kotsuji F, Winters SJ, Attardi B, Keeping HS, Oshima H, Troen P. Effects of gonadal steroids on gonadotropin secretion in males: studies with perifused rat pituitary cells. Endocrinology. 1988;123(6): 2683–9. 3143541 10.1210/endo-123-6-2683

[12] Starzec AB, Lerrant Y, Berault A, Counis R. Testosterone inhibits the basal and gonadotropin-releasing hormone-stimulated synthesis and release of newly synthesized alpha- and lutropin (LH) beta-subunit but not release of stored LH in cultured rat pituitary cells. Biochimica et biophysica acta. 1996;1310(3): 348–54. 8599614 10.1016/0167-4889(95)00178-6

[13] Jorgensen JS, Nilson JH. AR suppresses transcription of the LHbeta subunit by interacting with steroidogenic factor-1. Mol Endocrinol. 2001;15(9): 1505–16. 11518799 10.1210/mend.15.9.0691

[14] Okada Y, Fujii Y, Moore JP Jr, Winters SJ. Androgen receptors in gonadotrophs in pituitary cultures from adult male monkeys and rats. Endocrinology. 2003;144(1): 267–73. 12488354 10.1210/en.2002-220770

[15] Scheithauer BW, Kovacs K, Zorludemir S, Lloyd RV, Erdogan S, Slezak J. Immunoexpression of androgen receptor in the nontumorous pituitary and in adenomas. Endocrine pathology. 2008;19(1): 27–33. 10.1007/s12022-007-9012-0 18228161

[16] O'Hara L, Welsh M, Saunders PT, Smith LB. Androgen receptor expression in the caput epididymal epithelium is essential for development of the initial segment and epididymal spermatozoa transit. Endocrinology. 2011;152(2): 718–29. 10.1210/en.2010-0928 21177831

[17] Rodriguez S, Sickles HM, Deleonardis C, Alcaraz A, Gridley T, Lin DM. Notch2 is required for maintaining sustentacular cell function in the adult mouse main olfactory epithelium. Developmental biology. 2008;314(1): 40–58. 18155189 10.1016/j.ydbio.2007.10.056 PMC2374763

[18] Wang Y, Martin JF, Bai CB. Direct and indirect requirements of Shh/Gli signaling in early pituitary development. Developmental biology. 2010;348(2): 199–209. 10.1016/j.ydbio.2010.09.024 20934421 PMC3200223

[19] Hebert JM, McConnell SK. Targeting of cre to the Foxg1 (BF-1) locus mediates loxP recombination in the telencephalon and other developing head structures. Developmental biology. 2000;222(2): 296–306. 10837119 10.1006/dbio.2000.9732

[20] De Gendt K, Swinnen JV, Saunders PT, Schoonjans L, Dewerchin M, Devos A, et al A Sertoli cell-selective knockout of the androgen receptor causes spermatogenic arrest in meiosis. Proceedings of the National Academy of Sciences of the United States of America. 2004;101(5): 1327–32. 14745012 10.1073/pnas.0308114100 PMC337052

[21] O'Hara L, Smith LB. Androgen receptor signalling in Vascular Endothelial cells is dispensable for spermatogenesis and male fertility. BMC research notes. 2012;5: 16 10.1186/1756-0500-5-16 22230795 PMC3275443

[22] Welsh M, Saunders PT, Atanassova N, Sharpe RM, Smith LB. Androgen action via testicular peritubular myoid cells is essential for male fertility. FASEB journal: official publication of the Federation of American Societies for Experimental Biology. 2009;23(12): 4218–30. 19692648 10.1096/fj.09-138347 PMC2812048

[23] O'Hara L, York JP, Zhang P, Smith LB. Targeting of GFP-Cre to the mouse Cyp11a1 locus both drives cre recombinase expression in steroidogenic cells and permits generation of Cyp11a1 knock out mice. PloS one. 2014;9(1): e84541 10.1371/journal.pone.0084541 24404170 PMC3880310

[24] Corker CS, Davidson DW. A radioimmunoassay for testosterone in various biological fluids without chromatography. Journal of steroid biochemistry. 1978;9(4): 373–4. 96306 10.1016/0022-4731(78)90634-9

[25] Tyndall V, Broyde M, Sharpe R, Welsh M, Drake AJ, McNeilly AS. Effect of androgen treatment during foetal and/or neonatal life on ovarian function in prepubertal and adult rats. Reproduction. 2012;143(1): 21–33. 10.1530/REP-11-0239 22016380 PMC3245827

[26] Smith LB, Milne L, Nelson N, Eddie S, Brown P, Atanassova N, et al KATNAL1 regulation of sertoli cell microtubule dynamics is essential for spermiogenesis and male fertility. PLoS genetics. 2012;8(5): e1002697 10.1371/journal.pgen.1002697 22654668 PMC3359976

[27] Srinivas S, Watanabe T, Lin CS, William CM, Tanabe Y, Jessell TM, et al Cre reporter strains produced by targeted insertion of EYFP and ECFP into the ROSA26 locus. BMC developmental biology. 2001;1: 4 11299042 10.1186/1471-213X-1-4 PMC31338

[28] Simanainen U, McNamara K, Gao YR, McPherson S, Desai R, Jimenez M, et al Anterior prostate epithelial AR inactivation modifies estrogen receptor expression and increases estrogen sensitivity. American journal of physiology Endocrinology and metabolism. 2011;301(4): E727–35. 10.1152/ajpendo.00580.2010 21750267

[29] Yu IC, Lin HY, Liu NC, Wang RS, Sparks JD, Yeh S, et al Hyperleptinemia without obesity in male mice lacking androgen receptor in adipose tissue. Endocrinology. 2008;149(5): 2361–8. 10.1210/en.2007-0516 18276764 PMC2329275

[30] Bartke A, Smith MS, Michael SD, Peron FG, Dalterio S. Effects of experimentally-induced chronic hyperprolactinemia on testosterone and gonadotropin levels in male rats and mice. Endocrinology. 1977;100(1): 182–6. 830537 10.1210/endo-100-1-182

[31] Nonomura M, Hoshino K, Harigaya T, Hashimoto H, Yoshida O. Effects of hyperprolactinaemia on reproduction in male mice. The Journal of endocrinology. 1985;107(1): 71–6. 4045355 10.1677/joe.0.1070071

[32] Freeman ME, Kanyicska B, Lerant A, Nagy G. Prolactin: structure, function, and regulation of secretion. Physiological reviews. 2000;80(4): 1523–631. 11015620 10.1152/physrev.2000.80.4.1523

[33] Smith L. Good planning and serendipity: exploiting the Cre/Lox system in the testis. Reproduction. 2011;141(2): 151–61. 10.1530/REP-10-0404 21084571

[34] Krutskikh A, De Gendt K, Sharp V, Verhoeven G, Poutanen M, Huhtaniemi I. Targeted inactivation of the androgen receptor gene in murine proximal epididymis causes epithelial hypotrophy and obstructive azoospermia. Endocrinology. 2011;152(2): 689–96. 10.1210/en.2010-0768 21084446 PMC3101806

[35] Raskin K, de Gendt K, Duittoz A, Liere P, Verhoeven G, Tronche F, et al Conditional inactivation of androgen receptor gene in the nervous system: effects on male behavioral and neuroendocrine responses. The Journal of neuroscience: the official journal of the Society for Neuroscience. 2009;29(14): 4461–70. 10.1523/JNEUROSCI.0296-09.2009 19357272 PMC6665718

[36] Smith JT, Dungan HM, Stoll EA, Gottsch ML, Braun RE, Eacker SM, et al Differential regulation of KiSS-1 mRNA expression by sex steroids in the brain of the male mouse. Endocrinology. 2005;146(7): 2976–84. 15831567 10.1210/en.2005-0323

[37] Wersinger SR, Haisenleder DJ, Lubahn DB, Rissman EF. Steroid feedback on gonadotropin release and pituitary gonadotropin subunit mRNA in mice lacking a functional estrogen receptor alpha. Endocrine. 1999;11(2): 137–43. 10709760 10.1385/ENDO:11:2:137

[38] Eddy EM, Washburn TF, Bunch DO, Goulding EH, Gladen BC, Lubahn DB, et al Targeted disruption of the estrogen receptor gene in male mice causes alteration of spermatogenesis and infertility. Endocrinology. 1996;137(11): 4796–805. 8895349 10.1210/endo.137.11.8895349

[39] Lindzey J, Wetsel WC, Couse JF, Stoker T, Cooper R, Korach KS. Effects of castration and chronic steroid treatments on hypothalamic gonadotropin-releasing hormone content and pituitary gonadotropins in male wild-type and estrogen receptor-alpha knockout mice. Endocrinology. 1998;139(10): 4092–101. 9751487 10.1210/endo.139.10.6253

[40] Gieske MC, Kim HJ, Legan SJ, Koo Y, Krust A, Chambon P, et al Pituitary gonadotroph estrogen receptor-alpha is necessary for fertility in females. Endocrinology. 2008;149(1): 20–7. 17947360 10.1210/en.2007-1084 PMC2194602

[41] Singh SP, Wolfe A, Ng Y, DiVall SA, Buggs C, Levine JE, et al Impaired estrogen feedback and infertility in female mice with pituitary-specific deletion of estrogen receptor alpha (ESR1). Biology of reproduction. 2009;81(3): 488–96. 10.1095/biolreprod.108.075259 19439729 PMC2731984

[42] Temple JL, Scordalakes EM, Bodo C, Gustafsson JA, Rissman EF. Lack of functional estrogen receptor beta gene disrupts pubertal male sexual behavior. Hormones and behavior. 2003;44(5): 427–34. 14644637 10.1016/j.yhbeh.2003.09.002

[43] Garcia Barrado MJ, Blanco EJ, Carretero Hernandez M, Iglesias Osma MC, Carretero M, Herrero JJ, et al Local transformations of androgens into estradiol by aromatase P450 is involved in the regulation of prolactin and the proliferation of pituitary prolactin-positive cells. PloS one. 2014;9(6): e101403 10.1371/journal.pone.0101403 24978194 PMC4076335

[44] Ishaq M, Schang AL, Magre S, Laverriere JN, Guillou A, Coudouel N, et al Rat Gnrhr promoter directs species-specific gene expression in the pituitary and testes of transgenic mice. J Mol Endocrinol. 2013;50(3): 411–26. 10.1530/JME-12-0231 23536650

[45] Wu S, Chen Y, Fajobi T, DiVall SA, Chang C, Yeh S, et al Conditional Knockout of the Androgen Receptor in Gonadotropes Reveals Crucial Roles for Androgen in Gonadotropin Synthesis and Surge in Female Mice. Mol Endocrinol. 2014: me20141154. 10.1210/me.2014-1154 PMC4179628 25157703

[46] Israel JM, Cabelguen JM, Le Masson G, Oliet SH, Ciofi P. Neonatal testosterone suppresses a neuroendocrine pulse generator required for reproduction. Nat Commun. 2014;5: 3285 10.1038/ncomms4285 24518793

[47] Gonzalez-Parra S, Argente J, Garcia-Segura LM, Chowen JA. Cellular composition of the adult rat anterior pituitary is influenced by the neonatal sex steroid environment. Neuroendocrinology. 1998;68(3): 152–62. 9733999 10.1159/000054361

[48] Gill-Sharma MK. Prolactin and male fertility: the long and short feedback regulation. International journal of endocrinology. 2009;2009: 687259 10.1155/2009/687259 20011060 PMC2778443

[49] Ben-Jonathan N, Hnasko R. Dopamine as a prolactin (PRL) inhibitor. Endocrine reviews. 2001;22(6): 724–63. 11739329 10.1210/edrv.22.6.0451

[50] Tong Y, Simard J, Labrie C, Zhao HF, Labrie F, Pelletier G. Inhibitory effect of androgen on estrogen-induced prolactin messenger ribonucleic acid accumulation in the male rat anterior pituitary gland. Endocrinology. 1989;125(4): 1821–8. 2791967 10.1210/endo-125-4-1821

[51] Chandrashekar V, Bartke A. Influence of endogenous prolactin on the luteinizing hormone stimulation of testicular steroidogenesis and the role of prolactin in adult male rats. Steroids. 1988;51(5–6): 559–76. 3242177 10.1016/0039-128x(88)90052-9

[52] Steger RW, Chandrashekar V, Zhao W, Bartke A, Horseman ND. Neuroendocrine and reproductive functions in male mice with targeted disruption of the prolactin gene. Endocrinology. 1998;139(9): 3691–5. 9724019 10.1210/endo.139.9.6209

[53] Binart N, Melaine N, Pineau C, Kercret H, Touzalin AM, Imbert-Bollore P, et al Male reproductive function is not affected in prolactin receptor-deficient mice. Endocrinology. 2003;144(9): 3779–82. 12933648 10.1210/en.2003-0409

[54] De Rosa M, Zarrilli S, Vitale G, Di Somma C, Orio F, Tauchmanova L, et al Six months of treatment with cabergoline restores sexual potency in hyperprolactinemic males: an open longitudinal study monitoring nocturnal penile tumescence. The Journal of clinical endocrinology and metabolism. 2004;89(2): 621–5. 14764772 10.1210/jc.2003-030852

[55] De Rosa M, Colao A, Di Sarno A, Ferone D, Landi ML, Zarrilli S, et al Cabergoline treatment rapidly improves gonadal function in hyperprolactinemic males: a comparison with bromocriptine. European journal of endocrinology / European Federation of Endocrine Societies. 1998;138(3): 286–93. 9539303 10.1530/eje.0.1380286

